# A New Sight of Influencing Effects of Major Factors on Cd Transfer from Soil to Wheat (*Triticum aestivum* L.): Based on Threshold Regression Model

**DOI:** 10.3390/ijerph191912363

**Published:** 2022-09-28

**Authors:** Zhifan Chen, Wencai Geng, Xingyuan Jiang, Xinling Ruan, Di Wu, Yipeng Li

**Affiliations:** 1 College of Geography and Environmental Science, Henan University, Kaifeng 475004, China or; 2 Henan Engineering Research Center for Control & Remediation of Soil Heavy Metal Pollution, Henan University, Kaifeng 475004, China; 3 School of Economics, Henan University, Jinming District, Kaifeng 475004, China

**Keywords:** threshold effects, bioaccumulation, wheat (*Triticum aestivum* L.), *Eutyic Cambisols*, soil properties, transfer prediction

## Abstract

Due to the high toxicity and potential health risk of cadmium (Cd), the influencing effects of major factors (like pH, OM, and clay, etc.) on Cd bioaccumulation and transfer from soil to crop grains are highly concerned. Multiple linear regression models were usually applied in previous literature, but these linear models could not reflect the threshold effects of major factors on Cd transfer under different soil environmental conditions. Soil pH and other factors on Cd transfer in a soil–plant system might pose different or even contrary effects under different soil Cd exposure levels. For this purpose, we try to apply a threshold regression model to analyze the effects of key soil parameters on Cd bioaccumulation and transfer from soil to wheat. The results showed that under different soil pH or Cd levels, several factors, including soil pH, organic matter, exchangeable Cd, clay, P, Zn, and Ca showed obvious threshold effects, and caused different or even contrary impacts on Cd bioaccumulation in wheat grains. Notably, the increase of soil pH inhibited Cd accumulation when pH > 7.98, but had a promotional effect when pH ≤ 7.98. Thus, threshold regression analysis could provide a new insight that can lead to a more integrated understanding of the relevant factors on Cd accumulation and transfer from soil to wheat. In addition, it might give us a new thought on setting regulatory limits on Cd contents in wheat grains, or the inhibitory factors of Cd transfer.

## 1. Introduction

Cadmium (Cd) is of human and environmental health concern, because an excessive dietary intake of Cd and its accumulation from plant-based food sources into human organs over a long time period can lead to kidney malfunction [1]. Soil Cd is partly derived from parent materials due to chemical weathering. As a contaminant, it also comes from anthropogenic activities such as mining, smelting, composts, phosphate fertilizer application, waste disposal, and vehicle exhausts [2]. Because soil Cd is easily accumulated by plants, the soil–plant–human transfer of Cd has been considered as a major pathway of human exposure to soil Cd [2,3]. Thus, assessing the health risk of Cd from plants grown in various soil types, and understanding the effects of soil properties on Cd uptake and transfer in the soil–plant system are of great importance for soil pollution prevention and mitigation.

Numerous studies have demonstrated that a number of factors affect Cd bioavailability in soils, including soil pH, organic matter (OM), cation exchange capacity (CEC), and crop plant cultivars [4]. Various statistical testing methods have previously been applied to understand how various soil properties impact on Cd transfer and uptake in soil. Adams et al. (2004) applied a multiple regression model to predict and analyze Cd concentration in wheat grain based on soil properties, which showed that soil total Cd and pH were the significant factors influencing the grain Cd concentrations [5]. Brus et al. (2005) used a multiple linear regression equation to model the transfer of Cd in the soil to the crop grain, in order to calculate probabilistic Cd content standards in soil derived from food quality standards [6]. Liu et al. (2015) constructed a bioaccumulation prediction model from the soil into wheat plants using a multiple linear regression, which showed that pH was the most important factor contributing to Cd uptake [7]. Viala et al. (2017) proposed a linear statistical model to predict Cd accumulation in durum wheat grain based on soil geochemical properties related to Cd availability in French agricultural soils [8]. In recent years, some researchers (2018, 2020) have also applied a multiple linear regression model to analyze Cd transfer in soil–vegetable (or rice) systems [9,10].

However, one important limitation of the linear regression model is that different groups of entities may have different behaviors in a specific environmental problem. For instance, soil pH and other factors on Cd transfer in soil–plant systems might pose different or even contrary effects under different soil Cd exposure levels, while traditional statistical models usually neglect or simplify such effects. Thus, it is an important question of how to separate these two groups of Cd exposure levels and of estimating the respective effects of major controlling factors. That is to say, the choice of a threshold value that can reasonably separate these two groups. The traditional method is that a threshold value is determined subjectively by the researcher. For example, Yang et al. (2013) divided the soil Cd concentrations into three levels [4] (control Cd: 0.14~0.26 mg kg^−1^; low Cd levels: 0.3 mg kg^−1^ when soil pH < 7.5, and 0.6 mg kg^−1^ for pH > 7.5; high Cd levels: 0.6 mg kg^−1^ when pH < 7.5, and 1.0 mg kg^−1^ for pH > 7.5) according to Soil Environmental Quality Standards of China (GB15618-1995) [11], and then used multiple stepwise regressions to develop three prediction models on the effects of pH on Cd transfer from soil to corn grain under different Cd levels. Here, the parameter estimation of the threshold value and statistical test is not carried out; thus, the result might be not reliable. The threshold regression (TR) model was introduced by Tong (1978, 1983) [12,13], and Tong is designed to resolve such a question [14]. Details of the TR are available in Tong (1990, 2011) [15,16] for a summary of the TR literature in statistics, and Hansen (2011) in econometrics [17]. The TR method has been applied in economics [18,19] and in the medical field [20,21,22]. Recently, this model has become an emerging application in environmental sciences. For instance, the study of Song et al. (2016) used a timescale decomposed threshold regression downscaling approach and achieved results for predicting the observed rainfall in the validation period of 1985–2006 [23]; further optimization of such a technique may provide a new perspective for improving climate predictions. Liu et al. (2022) established a threshold regression model with urbanization level as the threshold variable, revealing a nonlinear relationship between population structure and carbon emissions [24]. Feng et al. (2020) used threshold regression to conduct nonlinear analysis on different types of environmental regulation tools in Beijing Tianjin Hebei region, providing a basis for the government to implement policies related to environmental regulation tools [25]. In this study, we attempted to use the TR to provide a more precise identification and estimates of the effects of soil controlling factors on Cd transfer in the soil–crop system.

The alluvial plain area of the middle reaches of the Yellow River is one of the important agricultural production areas in China. The predominant soil type in this area is *Eutyic Cambisols* [26]. Wheat is one of the main crops grown in the area. In this study, a typical industry–agriculture transition area was chosen as the study area. Here, the total soil Cd concentrations and various soil properties have a greater variability, which provided ideal field conditions for studying the effects of various soil properties on Cd uptake by wheat plants. The objectives of the present study were to: (1) apply the threshold regression model to analyze the effects of soil properties (including soil pH, organic matter, exchangeable Cd, clay, P, Zn and Ca, etc.) on the transfer of Cd from soil to wheat grain in the Yellow River alluvial plain; (2) identify the threshold effects of major soil factors under different soil Cd or pH levels, and to provide a new insight on an understanding of relevant factors on Cd accumulation and transfer from soil to wheat.

## 2. Materials and Methods

### 2.1. Description of the Study Area

**Figure 1:**
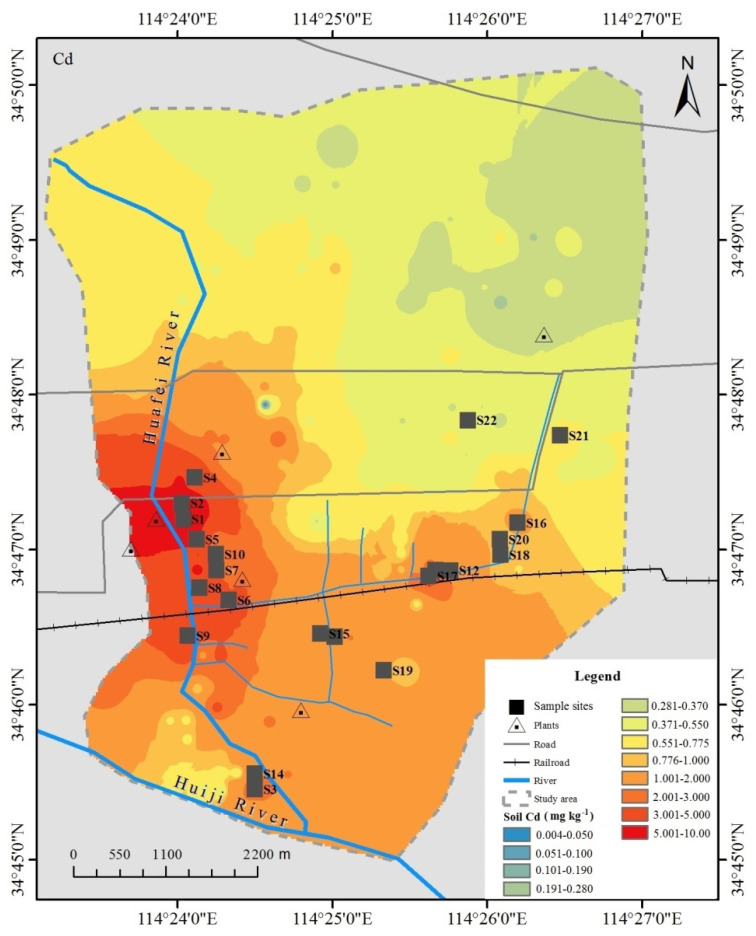
The paired soil–wheat sample sites and study area (adapted from our previous study: Chen et al., 2016).

The study area is approximately 20 km^2^ in size, and is located in the eastern sub-urban area of Kaifeng city, Henan Province (the Yellow River alluvial plain), which is an important agricultural production zone of China (Figure 1). This area has a continental monsoon climate, characterized by a wide seasonal variation in rainfall (annual 627.5 mm), with cold and dry winters, and hot and rainy summers. According to the United Nations soil classifications, *Eutyic Cambisols* is the predominant soil type in this area, which was developed from the Yellow River alluvial deposits [26]. Wheat is one of the important crops grown in the area. Industrial and mining enterprises, including chemical fertilizer plants, zinc smelting plants, a carbon factory, pharmaceutical companies, and a thermal power plant, existed in the study area. The Huafei River, running from north to south through the study area, receives wastewater from these industrial sources. The Huafei River had been used for agricultural irrigation in the area since 1962. Thus, the study area presented a coexistence state of sewage irrigation and industrial and agricultural pollution, which has led to the accumulation of heavy metals (especially Cd) in the soil–crop system. As shown in Figure 1, the total Cd contents in the studied soils varied from 0.42 to 11.21 mg kg^−1^, which showed a gradient distribution (from unpolluted to low and high polluted levels) centering on the pollution sources [27].

### 2.2. Sampling and Preparation

A total of 22 samples, each consisting of topsoil and wheat plant (*Triticum aestivum* L.) samples were collected from the study area. At each sample site, five topsoil (depth, 0–20 cm) subsamples were gathered in a 10 × 10 m^2^ area. Sample locations are shown in Figure 1. At the same time, five corresponding wheat plant subsamples were collected and combined into a single composite sample (NY/T 1121.1-2006) [28]. The soil samples were air-dried and milled with a porcelain mortar to pass through a 0.149 mm pore size nylon sieve [29,30]. The plant samples were carefully rinsed with deionized water, initially air-dried at room temperature, and ground into fine particles. The powered samples of plant tissues were oven-dried at 65 °C for 48 h prior to chemical analysis [29].

### 2.3. Analytical Methods

Soil properties were measured using routine methods [29]. Soil pH was measured in a 2.5:1 water/soil suspension using a pH meter; calcium carbonate, CaCO_3_, content was determined using the gas-volumetric method [29]; soil organic matter (OM) content was measured with the K_2_Cr_2_O_7_-H_2_SO_4_ oil-bath-heating digestion method [29]. The particle size of soil samples was analyzed using a Mastersizer 2000 laser particle size analyzer (Malvern, UK) after pretreatment with 10% H_2_O_2_, 10% HCl, and 0.05 mol L^−1^ (NaPO_3_)_6_ dispersant (HJ 1068-2019) [31]. According to soil texture classification in the United States, the soil particles were divided into clay, silt, and sand.

A total of 0.1000 g of each dried soil sample was digested with a 5 mL HNO_3_-HClO_4_-HF (3:1:1, *v*/*v*/*v*) mixture [30] using the full automatic graphite digestion instrument (ST-60, Polytech Instrument Ltd., Beijing, China), and then the concentrations of total Fe, Mn, K, and P in the solution were determined using inductively coupled plasma atomic emission spectrometer (ICP-AES, Thermo iCAP 6000 series, Waltham, MA, USA). Cd concentrations were determined via inductively coupled plasma mass spectrometry (ICP-MS, Thermo Fisher X-Series II, Waltham, MA, USA).

A total of 0.5000 g of each homogenized composite wheat grain sample was weighed and digested with a 10 mL mixture of HNO_3_: HClO_4_ (4:1, *v*/*v*) using a full automatic graphite digestion instrument (ST-60, Polytech Instrument Ltd., Beijing, China). The digests were diluted with deionized water to a 100 mL volumetric flask, and to a final volume of 100 mL. The concentrations of Cd in the solutions were determined using ICP-MS.

The sequential extraction scheme proposed by Tessier et al. (1979) was adopted to separate Cd into five fractions: exchangeable (ex-Cd), bound to carbonates (ca-Cd), bound to Fe-Mn oxides (FM-Cd), bound to organic matter (or-Cd), and residual (re-Cd). Specific sequential extraction was referenced in the previous literature [32,33].

Duplicates, method blanks, and standard reference materials were analyzed. The certified reference soil and wheat materials were GSS-2 and GBW10011, respectively, from the National Research Center for Standard Materials, China. Mean recoveries for Cd in soil and plant samples were within 100 ± 5% of the standard material recoveries. The method blanks in each batch of samples were negligible. The mean value of percentage recovery for sequential extraction was 95 ± 5%.

### 2.4. Data Analysis

#### 2.4.1. Bioconcentration Factor (BCF)

The bioconcentration factor (BCF) was defined as the ratio of Cd concentration (mg kg^−1^ dry wt) in the plants to the Cd concentration determined in the corresponding soils [34]. BCF was used to evaluate the Cd accumulation ability of plants.

#### 2.4.2. Threshold Regression Analysis

Our hypothesis is that the controlling factors have different effects on Cd accumulation and transfer in wheat under different soil Cd contents or soil pH. Splitting the variable point was based off the threshold regressions of Hansen (2000) [35]. Following Hansen (2000), the following model was first established, in which the relationship between the independent variable *x_i_* and the dependent variable y*_i_* is driven by one single threshold variable:

(1) $$y_{i}=\{\begin{array}{c} \beta_{1}x_{i}+\varepsilon_{i},q_{i}\leq \gamma \\ \beta_{2}x_{i}+\varepsilon_{i},q_{i}>\gamma \end{array}$$

where y*_i_* is the dependent variable (here, it is log[BCF]), *x_i_* is the exogenous explanatory variable (here, it is controlling factors, such as pH, OM, etc.), *q_i_* is defined as the threshold variable used to split the sample (here, it is soil Cd contents or soil pH), *γ* denotes the threshold point, and *β*_1_, *β*_2_, *ε_i_* are unknown parameters to be estimated. Two regimes are defined in the above setting: regime 1 is set, where *q_i_* ≤ *γ*, while regime 2 is defined where *q_i_* > *γ*.

One of the difficulties in the operating threshold models is the estimation of threshold parameters. In this study, the main controlling factors and their effects under different Cd and pH levels were analyzed and predicted based on threshold regression method developed by Hansen (1996, 2000) using Stata14.1 [35,36].

#### 2.4.3. Statistical Analysis

Statistical analysis was performed using PASW Statistics 18 for Windows. Correlation analysis was performed using the Pearson’s correlation procedure. The statistical significance of grouped means differences was computed using one-way ANOVA, and data with *p* < 0.05 were considered significant. Figures were produced using Origin Pro 8.0.

## 3. Results

### 3.1. Cd Concentration and Soil Properties

Data comprising a summary of the 22 soil samples are described in Table 1, and include the total soil Cd concentration and various soil properties (including pH, OM, and particle sizes). The total Cd contents in the studied soils ranged from 0.42 to 11.21 mg kg^−1^, and their mean value (2.99 mg kg^−1^) exceeded the background value of soils for *Eutyic Cambisols*, China (0.10 mg kg^−1^ (National Environmental Monitoring Station of China, 1990) [37]. Meanwhile, the coefficient of variation (CV) for total soil Cd concentrations reached 89.40%, implying that the existence of high concentrations of Cd in soil may be derived from external sources such as industrial emissions, automobile exhaust, and farming practices. Compared with soil environmental quality standards in China, 77.27% of the soil samples exceeded the maximum allowable concentration of Cd in agricultural soils (1 mg kg^−1^) (National Standard of PR China, 1995) [11].

The studied soils were developed from the Yellow River alluvial deposits, containing high concentrations of CaCO_3_ (43.32~106.07 g kg^−1^). Soil pH ranged over 6.82~8.78, showing a neutral to mild alkaline feature of the studied soils. The mean value of OM was 3.02%. For the composition of the soil particle sizes, there were very low clay contents (0~2 μm) with an average value of 1.20%, while silt and sandy particles accounted for higher percentages, with their average contents of 67.80% and 31.00%, respectively. The average concentrations of the elements Ca, Fe, Mn, P, and Zn were 31.78 g kg^−1^, 30.11 g kg^−1^, 0.57 g kg^−1^, 1.23 g kg^−1^, and 488.44 mg kg^−1^, respectively. It was observed that the P and Zn concentrations for studied soils had high CVs (21.58% and 171.28%, respectively), implying potential anthropogenic sources of these elements, such as from industrial emissions and the excessive application of fertilizers. The wide range of total soil Cd concentrations from 0.42 to 11.21 mg kg^−1^, together with the great spatial variability of soil properties, provided ideal information for studying the effects of various soil properties on Cd uptake by wheat plants under field conditions.

### 3.2. Chemical Speciation of Cd under Sequential Extraction

**Figure 2:**
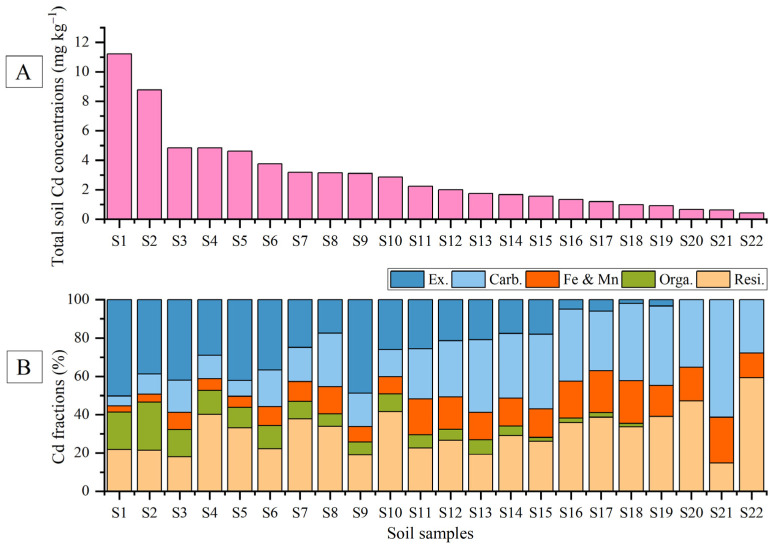
The concentrations of total Cd (**A**), and the percentages of each Cd fraction (**B**) in soil samples.

Figure 2 shows the total Cd contents and the percentages of Cd fractions in each soil sample. The average concentrations of Cd in the different fractions were generally in the order of re-Cd (38%) > ca-Cd (30%) > FM-Cd (15%) > ex-Cd (13%) > or-Cd (4%). According to the previous literature [33], the non-residual fractions (ex-Cd, ca-Cd, FM-Cd, and or-Cd) represent the activity of heavy metal. The sum of the non-residual fractions of Cd in the studied soils accounted for about 62% of the total soil Cd, suggesting that Cd in the soil samples have high mobility and bioavailability. Totally, with the decrease of total Cd in the soils, the percentages of the major bioavailable fractions, such as ex-Cd and or-Cd, presented decreasing trends.

### 3.3. Cadmium Accumulation in Wheat Grains, and Correlation with Soil Parameters

**Figure 3:**
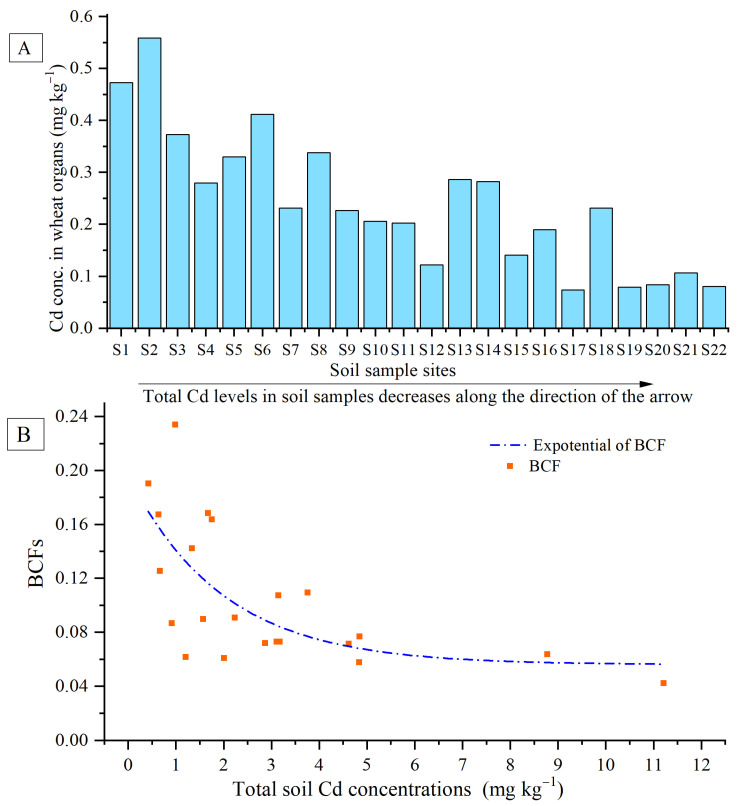
Total Cd concentrations in wheat grains (**A**), and the wheat grain BCFs under different soil Cd levels (**B**).

The concentrations of Cd in wheat grain and their BCFs are presented in Figure 3. Generally, with increasing total soil Cd levels, Cd concentrations in grain showed increasing trends. According to the maximum permissible concentrations (MPC) for Cd in wheat grains (0.1 mg kg^−1^) (GB 2762-2017) [38], 81.82% of the wheat grain samples exceeded this MPC, demonstrating the health potential risks of Cd in wheat grains. Correlation coefficients between Cd concentrations in wheat grain and relevant soil properties are listed in Table 2. Cd accumulation in wheat grain exhibited significant positive correlations with total soil Cd, soil P, soil Zn, and 50 μm soil particle size, but significant negative correlations with pH and 2~50 μm particle size.

Cd accumulation in wheat grains can be evaluated using BCF (the ratio of Cd grain concentration/Cd concentration in soil). Under field conditions, wheat plants had lower BCFs, with a range of 0.04~0.23. With increasing soil Cd concentrations up to 11.21 mg kg^−1^, BCF values declined slowly and gradually stabilized with very low BCF values of around 0.05. Furthermore, it was found that BCF and the log[BCF] of wheat grains showed highly significant negative correlations (*p* < 0.01) with total soil Cd, Ex-Cd, soil P, and soil Zn. Meanwhile, a significant positive correlation (*p* < 0.05) could be observed between grain BCF and soil clay contents, log[BCF], and pH, and a significant negative correlation (*p* < 0.05) between grain log[BCF] and log[OM] was also observed.

### 3.4. Threshold Effects of Main Soil Factors under Different Soil Cd and pH Levels

**Figure 4:**
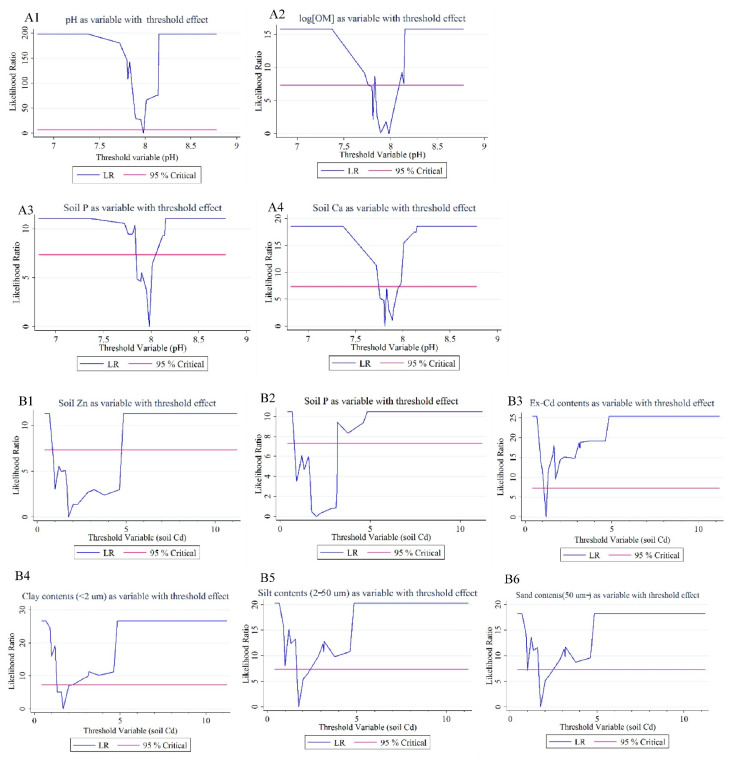
Likelihood ratio (LR) sequence of several variables (pH, log[OM], soil P, soil Ca, Ex-Cd, clay, and silt and sand), with threshold effects for pH or total soil Cd levels as threshold variable.

Threshold regression analysis can reflect the threshold effects of key soil factors on Cd accumulation and a transfer from soil to wheat under varying soil Cd or pH levels. Based on the threshold regression method developed by Hansen [35,36], the threshold effects of the soil factors were analyzed under different pH and total soil Cd levels. The threshold estimate values, predication models, and likelihood ratio sequences of several variables (such as soil pH, log[OM], soil P, soil Ca, soil Zn, ex-Cd, clay, and silt and sand contents), with threshold effects with pH or total soil Cd levels as the threshold variable, are shown in Table 3 and Figure 4. Under different pH levels, the threshold effects of soil factor pH, log[OM], soil P, and soil Ca on log[BCF] of Cd in a soil–wheat system were significant. Here, pH was threshold variable and its threshold estimate value was 7.98 (soil Ca is an exception) (Table 3: (1)–(4) and Figure 4(A1–A4)). When pH > 7.98, the value of log[BCF] decreased with the increase in pH value (Table 3: (1)). However, when pH ≤ 7.98, the opposite effect happens, and the value of log[BCF] increased with the increase in pH value. In addition, as log[OM] increased, log[BCF] decreased, which was similar to the study of Liu et al. (2015) [39]. Furthermore, the inhibitory effect of OM on Cd accumulation was more remarkable under a weaker alkaline condition. Soil P also had inhibitory effects on Cd accumulation, and the inhibitory effect was stronger under higher alkaline conditions. Soil Ca had a similar inhibitory effect on Cd accumulation. However, the total soil Cd, Ex-Cd, and nonResi-Cd showed no threshold effect, with pH as the threshold variable. The R-squared values or parameters of prediction models of soil Fe, Mn, OM, CaCO_3_, and soil Zn were very small.

Under different total soil Cd levels, the threshold regression analysis of the ex-Cd fraction on Cd accumulation in the soil–wheat system was conducted, as shown in Table 3 (7). Figure 4(B3) shows the likelihood ratio sequence, threshold estimate value, and prediction models of Ex-Cd concentrations. When total soil Cd levels were below 1.20 mg kg^−1^, the increase in Ex-Cd concentrations had a strong inhibition on Cd accumulation. With the increase in Ex-Cd concentrations, BCF values declined sharply. When total soil Cd levels were above 1.20 mg kg^−1^, the increase in Ex-Cd concentrations had a weak inhibition on Cd accumulation. With the increase in Ex-Cd concentrations, BCF values declined slowly and tended to be stable. However, other Cd speciation in soils, including Carb-Cd, Fe/Mn-Cd, Orga-Cd, and nonResi-Cd, had no significant threshold effects.

Table 3 (8)–(10) and Figure 4 (B4–B6) show the threshold estimate values, prediction models, and likelihood ratios of different soil particles, including clay content (<2 μm), silt content (2~50 μm), and sand content (>50 μm), with soil Cd as the threshold variable. Under different total soil Cd levels, different soil particle sizes on log [BCF] of Cd in a soil–wheat system have significant threshold effects, and the threshold estimate values of total soil Cd as a threshold variable were 1.75, except for clay contents (1.67). When soil Cd levels were below 1.67 mg kg^−1^, clay content (<2 μm) had significant positive effects on Cd accumulation. The increase in clay content accelerates Cd accumulation in wheat. However, when soil Cd levels were above 1.67 mg kg^−1^, clay content imposed significant inhibitory effects on Cd accumulation. Therefore, increasing the clay content in soil would decelerate Cd accumulation in wheat. This result is different from that of correlation analysis. Similar to the clay contents, silt contents (2~50 μm) and sand contents (>50 μm) showed positive effects on Cd accumulation in soil when soil Cd levels were below 1.75 mg kg^−1^, and inhibited accumulation when soil Cd levels were above 1.75 mg kg^−1^.

## 4. Discussions

### 4.1. Effects of pH, Ex-Cd, and OM on Cd Uptake, and Transfer from Soil to Wheat

In the previous literature, many researchers studied the effect of soil pH on Cd transfer from soil to plant, based on traditional statistical methods, such as linear fitting and multiple regressions [4,5,6,7,8,40]. These studies showed that Cd accumulation in wheat grains was negatively correlated with pH levels, and with the increment of soil pH levels, Cd accumulation in plants would be inhibited (Table 4). The reason for this might be that the pH affects the partitioning of metals between the soil phase and the aqueous phase, and the response in plant roots to the uptake of ions. Increasing the pH favors the adsorption of Cd to metal binding sites, and decreases the partition of Cd to the soil solution [41,42]. In our study, when soil pH levels were above 7.98, Cd bioaccumulation in wheat grain was inhibited with the increment of soil pH levels, and Cd accumulation in wheat grain was promoted with the decrease in soil pH levels (Table 3), which was good and consistent with the previous literature. However, notably, when the soil pH levels were below 7.98, the increment of pH promoted Cd uptake and transfer, while the decrease in pH inhibited Cd accumulation, which was different from the previous studies. The reason for this might be that the wheat growth in the study area needs an optimum soil pH interval. The soil in an acidic or neutral state might pose disadvantageous effects on the normal growth of wheat, and Cd accumulation was also inhibited. Thus, based on the threshold regression analysis, it was observed that under different soil pH levels, the changes of pH might have different or even contrary effects on Cd bioaccumulation. The result gave us a new insight in that the effects of soil pH on Cd bioaccumulation are not a simple linear negative correlation, and the optimum intervals of Cd bioaccumulation should be considered when considering phytoremediation and the establishment of soil environmental quality standards.

Soil ex-Cd was considered to be a primarily bioavailable form for plants [43]. With an increase in ex-Cd concentrations in soils, Cd concentrations in wheat grains increased, while the BCF values of wheat grain declined. Furthermore, threshold regression analysis demonstrated that under different total soil Cd levels, ex-Cd concentrations showed obvious threshold effects on Cd bioaccumulation. When total soil Cd levels were below 1.20 mg kg^−1^, with the increase in ex-Cd concentrations, BCF values declined sharply. It was indicated that the increasing ex-Cd concentrations showed a strong inhibition on Cd accumulation. When total soil Cd levels were above 1.20 mg kg^−1^, BCF values declined slowly and tended to be stable with the increase in ex-Cd concentrations. The inhibition of the increasing ex-Cd on the increment of Cd bioaccumulation became weak (Table 3). This result was well consistent with scatter modeling and previous studies. For instance, the study of Garrett et al. (1998) showed that extractable Cd and organic C could explain 74% of the variability of grain Cd (n = 34) [44]. Gray et al. (2001) reported that soil total Zn and extractable Cd together explained 59% of the variability of Cd in wheat grains in New Zealand [45]. Norvell et al. (2000) found that extractable Cd in soil accounted for 66% of the variability of Cd in grains [46]. However, other speciation of Cd in soils, including ca-Cd, FM-Cd, or-Cd and nonResi-Cd, had no significant threshold effects (data are not listed in this study). It was further confirmed that ex-Cd was a key bioavailable fraction [43], and that its concentration played major impacts on Cd bioaccumulation.

Soil organic matter (OM) also exhibited a certain influence on the transfer and availability of Cd in plants [6,39]. Our correlation analysis showed that the soil organic matter content had no obvious correlation with Cd bioaccumulation in wheat grains, while there was a significant negative correlation between log[OM] and log[BCF]. Threshold regression analysis also demonstrated that under different pH levels, log[OM] showed significant negative correlation with log[BCF], agreeing with previous research [39]. This result indicated that with an increasing organic matter content, Cd uptake and transfer in the soil–wheat system would be restricted.

### 4.2. Effects of Several Main Elements on Cd Uptake and Transfer from Soil to Wheat

There is a common transport site and transport process between Cd and other elements like Zn, Ca, Fe, Mn, and P. These elements usually show antagonistic effects on Cd uptake and accumulation through binding to the thiol group in an enzyme or protein responsible for nutrient uptake [47]. For instance, the study of Wang et al. (2016) showed that Cd and Zn interact antagonistically in wheat roots, and that Cd uptake was inhibited by Zn [48]. In our study, there was a significant positive correlation between soil Zn and grain Cd, while a negative correlation existed between soil Zn and BCF, and soil Zn and log[BCF] (Table 2). This demonstrated that Zn posed an antagonistic effect on Cd accumulation. Moreover, under different soil Cd levels, the inhibition of Zn on Cd accumulation showed threshold effects. When soil Cd levels were below 1.75 mg kg^−1^, Zn presented a stronger antagonistic effect than that of soil Cd levels of above 1.75 mg kg^−1^. The previous research showed that as an important micronutrient, Zn was also an antagonist to Cd, which could limit Cd entry into the food chain [49]. The research of Saifullah et al. (2014) showed that the foliar application of Zn at a suitable concentration can effectively ameliorate the adverse effects of Cd exposure and decrease the grain Cd concentration of wheat grown in Cd-contaminated soils [50]. In addition, no clear threshold effect was found under different pH conditions.

Soil P and Ca showed significant antagonistic effects and threshold effects on Cd uptake and transfer under different pH conditions (Table 3: (3)–(4)). When the pH was above 7.98 (for soil P) or 7.81 (for soil Ca), increasing soil Ca and P showed stronger inhibitory effects on Cd accumulation than that of pH below 7.98 or 7.81. In addition, under different soil Cd levels, soil P showed clear threshold effects on Cd accumulation. When soil Cd levels were below 2.01 mg kg^−1^, soil P presented stronger antagonistic effects on Cd accumulation than those of soil Cd levels above 2.01 mg kg^−1^.

### 4.3. Impacts of Different Soil Particle Sizes on Cd Uptake and Transfer from Soil to Wheat

Soil texture is one of the important factors impacting upon rhizosphere environment and metals migration in the field [43]. Existing research showed that clay had the strongest adsorption and retention ability to Cd in soils, followed by loam and then sandy soil. For example, the study of Shao et al. (2012) showed that Cd stress to wheat plants was alleviated in clay and loam, due to less Cd mobility than in sandy soil, and Cd accumulation for grown wheat was less in loam and clay than in sandy soil [43]. The study of Zhang et al. (2012) also showed that the adsorption of various soil particles to Cd were in the order of clay > silt > sand [51]. In our study, a correlation analysis showed that soil clay and silt particle contents were negatively correlated with Cd concentrations in wheat grains (significant for silt), and sand particle contents were positively correlated with Cd accumulation in wheat grains, as shown in Table 3. This result agrees with previous studies, providing further confirmation that a stronger adsorption on Cd of clay and silt inhibited Cd uptake by wheat plants. However, it was noticed that there was a significant positive correlation between BCF and soil clay contents (*p* < 0.05), but no significant positive correlation existed between BCF and soil silt contents, and no negative correlation was between BCF and sand contents (Table 3: (8)–(9)). A similar correlation existed between log[BCF] and different soil particle sizes. This result implied that the impacts of soil particle contents on Cd accumulation and transfer in a soil–wheat system might be closely related with soil Cd levels.

According to Table 3: (8)–(9), under different soil Cd levels, different soil particle contents posed different impacts on Cd uptake and transfer in a soil–wheat system. When soil Cd levels were below 1.67 mg kg^−1^, increasing the percentage of clay particles in soil played promoting effects on Cd accumulation in wheat plants. The reason for this might be that the Cd desorption by soil clay particles was higher than that of the absorption when the soil Cd concentrations were low. However, when soil Cd levels were above 1.67 mg kg^−1^, the increase in soil clay particle contents presented significant inhibitory effects on Cd accumulation. It was also found that the equilibrium of Cd absorption and desorption by soil silt and sand particles had threshold effects that were similar to soil clay particle (the threshold estimate values were 1.75); however, both the promoting and inhibitory effects seemed to be very weak, according to their parameters and R^2^ values.

## 5. Conclusions

Due to carcinogenicity and high health risk, Cd uptake and transfer in the soil–crop system is a concern. In this study, threshold regression analysis was conducted to analyze the effects of major environmental factors on Cd bioaccumulation in wheat grains. Our research found that under different pH or soil Cd levels, several major factors, including pH, log[OM], ex-Cd, soil clay contents, soil P, soil Zn, and soil Ca showed obvious threshold effects, and posed different or even contrary impacts on Cd bioaccumulation in wheat grains. Especially, when soil pH > 7.98, the rise in soil pH would inhibit Cd accumulation in wheat grains, while presenting a promotional effect when pH ≤ 7.98. However, these different effects cannot be identified through traditional linear regression models. It can be seen that threshold regression analysis gives us a new view, leading to a more comprehensive understanding on the influencing factors and effects of Cd accumulation and transfer in a soil–wheat system. In addition, it was suggested that a reasonable interval of key factors need be considered when the maximum allowable concentrations of Cd in soil are formulated, or when the inhibitory technologies of Cd transfer are adopted. Certainly, a large number of investigation practices and experimental simulation analyses will be still necessary for determining the optimal intervals.

## Figures and Tables

**Table 1:** Statistics of Cd concentrations and soil properties (n = 22).

	Mean	Median	Min.	Max.	CV
Soil Cd (mg kg^−1^)	2.99 ± 2.67	2.12	0.42	11.21	89.40%
pH	7.88 ± 0.38	7.90	6.82	8.78	4.82%
CaCO_3_ (g kg^−1^)	67.86 ± 18.79	72.79	43.32	106.07	27.69%
OM (%)	3.02 ± 0.56	2.93	2.26	4.08	18.60%
Clay, 0–2 μm (%)	1.20 ± 0.39	1.10	0.75	2.08	32.39%
Silt, 2–50 μm (%)	67.80 ± 7.14	69.48	51.96	78.82	10.54%
Sand, 50 μm (%)	31.00 ± 7.33	29.48	19.11	46.97	23.64%
Soil Ca (g kg^−1^)	31.78 ± 6.10	31.61	22.52	42.93	19.19%
Soil Fe (g kg^−1^)	30.11 ± 2.87	29.69	26.17	38.14	9.52%
Soil Mn (g kg^−1^)	0.57 ± 0.05	0.56	0.50	0.72	9.08%
Soil P (g kg^−1^)	1.23 ± 0.26	1.16	0.93	1.77	21.58%
Soil Zn (mg kg^−1^)	488.44 ± 836.58	198.96	110.64	4057.14	171.28%

**Table 2:** Correlation coefficients between Cd and related parameters in the soil–wheat system.

	Grain Cd	BCF	Log[BCF]
Soil Cd	0.847 **	−0.588 **	−0.661 **
Ex-Cd	0.802 **	−0.616 **	−0.675 **
Soil Ca	−0.206	0.331	0.291
Soil Fe	0.283	−0.017	−0.023
Soil Mn	−0.182	−0.025	−0.065
Soil P	0.357	−0.669 **	−0.726 **
Soil Zn	0.586 **	−0.428 *	−0.541 **
pH	−0.534 *	0.363	0.426 *
CaCO_3_	−0.118	0.295	0.237
OM	−0.230	−0.381	−0.409
Log[OM]	−0.219	−0.403	−0.427 *
Clay (<2 μm)	−0.374	0.472 *	0.408
silt (2–50 μm)	−0.593 **	0.298	0.323
Sand (>50 μm)	0.598 **	−0.316	−0.337

** Correlation is significant at the 0.01 level (two tailed); * Correlation is significant at the 0.05 level (two-tailed).

**Table 3:** Predication Models for the key soil factors under the different pH levels or Cd levels, based on threshold regression (n = 22, *p* ≤ 0.05).

Model No.	Controlling Factor	Threshold Estimate	Prediction Models	R^2^
(1)	pH	7.98	Log[BCF] = −3.337 + 0.292 pH pH ≤ 7.98	0.354
			Log[BCF] = 4.186 − 0.615 pH pH > 7.98	0.500
(2)	Log[OM]	7.98	Log[BCF] = −0.608 − 0.998 log[OM] pH ≤ 7.98	0.321
			Log[BCF] = −0.496 − 0.831 log[OM] pH > 7.98	0.066
(3)	Soil P	7.98	Log[BCF] = −0.606 − 0.375 [soil P] pH ≤ 7.98	0.488
			Log[BCF] = 0.252 − 1.008 [soil P] pH > 7.98	0.766
(4)	Soil Ca	7.81	Log[BCF] = −0.926 − 0.008 [soil Ca] pH ≤ 7.81	0.204
			Log[BCF] = −1.325 − 0.012 [soil Ca] pH > 7.81	0.155
(5)	Soil Zn	1.75	Log[BCF] = −0.698 − 0.001 [soil Zn] soil Cd ≤ 1.75	0.037
			Log[BCF] = −1.081 − 0.0001 [soil Zn] soil Cd > 1.75	0.504
(6)	Soil P	2.01	Log[BCF] = 0.217 − 1.044 [soil P] soil Cd ≤ 2.01	0.557
			Log[BCF] = −0.759 − 0.272 [soil P] soil Cd > 2.01	0.412
(7)	Ex-Cd	1.20	Log[BCF] = −0.761 − 3.898 [ex-Cd] soil Cd ≤ 1.20	0.509
			Log[BCF] = −0.911 − 0.245 [ex-Cd] soil Cd >1.20	0.490
(8)	Clay (<2 μm)	1.67	Log[BCF] = −1.205 + 0.219 [Clay] soil Cd ≤ 1.67	0.309
			Log[BCF] = −0.366 − 0.731 [Clay] soil Cd > 1.67	0.739
(9)	Silt (2–50 μm)	1.75	Log[BCF] = −0.685 + 0.003 [Silt] soil Cd ≤ 1.75	0.013
			Log[BCF] = −1.537 − 0.006 [Silt] soil Cd > 1.75	0.091
(10)	Sand (>50 μm)	1.75	Log[BCF] = −0.928 + 0.002 [Sand] soil Cd ≤ 1.75	0.007
			Log[BCF] = −0.948 − 0.006 [Sand] soil Cd > 1.75	0.078

**Table 4:** Literature comparisons of soil factors and prediction models for Cd accumulation and transfer in a soil–wheat system.

Considered Controlling Factor	Prediction Models	R^2^	*p*	Conditions	Literature
**pH, Lg[Cd_total_]**	Lg[Cd_grain_] = 0.28 + 0.44 Lg[Cd_total_] − 0.18 pH	0.42	<0.05	N = 162, pH 5.2–8.3	[5]
**pH, Lg[Cd_total_], Lg[SOM]**	Lg[Cd_grain_] = 1.022 + 0.749 Lg[Cd_total_] − 0.257 pH − 0.277 Lg[SOM]	0.44	<0.05	N = 84, pH 4.4–7.4	[6]
pH, control Cd *	Lg[BCF] = −0.081 pH − 0.254	0.728	<0.001	N = 17, pH = 4.90–8.80, OM = 8.57–47.69 g kg^−1^	[4]
pH, low Cd *	Lg[BCF] = −0.104 pH − 0.170	0.811	<0.001		
pH, high Cd *	Lg[BCF] = −0.079 pH − 0.280	0.713	<0.001		
**pH, Lg[Cd_total_]**	Lg[Cd_grain_] = 1.386 + Lg[Cd_total_] − 0.279 pH	0.85	<0.001	N = 14, pH 5.74–8.37, OC 6.78–27.66 g kg^−1^	[7]
**pH, Lg[Cd_total_]**	Lg[Cd_grain_] = 0.703 + 1.04 Lg[Cd_total_] − 0.175 pH	0.61	<0.001	N = 99, pH 5.0–8.6, OM 8.49–57.9 g kg^−1^	[41]
**pH, LgCsoil**	LgC_grain_ * = −0.257 pH + 1.203 + LgC_soil_ *	0.85	<0.001	N = 14, pH 5.74–8.65, OC 4.97–27.7 g kg^−1^	[40]
**pH, Lg[OC], LgCsoil**	LgC_grain_ = −0.280 pH − 0.446Lg[OC] * + 1.848 + LgC_soil_	0.93	<0.001		
pHCaCl_2_ *, Lg[Cd_DGT_ *]	Lg[Cd_grain_] = 7.359 + 0.697 Lg[Cd_DGT_] − 1.014 pHCaCl_2_	0.66	<0.001	N = 26, pH = 8.0–8.7, OC 9.21–40.3 g kg^−1^	[8]

* Control Cd means the background levels of soil Cd with the range of 0.14–0.26 mg kg^−1^. Low Cd levels were 0.3 mg kg^−1^ when soil pH < 7.5, and 0.6 mg kg^−1^ for pH > 7.5. High Cd levels were 0.6 mg kg^−1^ when pH < 7.5, and 1.0 mg kg^−1^ for pH > 7.5. LgBAF = Log[C_plant_] − Log[C_soil_]; Cgrain means Cd concentrations in wheat grain; Csoil means Cd concentrations in soil; [OC] means organic carbon contents in soil. SOM is soil organic matter concentration in %. pHCaCl_2_ means the pH measured in the CaCl_2_ extract. Cd_DGT_ means Cd from the DGT [8].

## Data Availability

It is excluded.
